# Used protocols for isolation and propagation of ovarian stem cells, different cells with different traits

**DOI:** 10.1186/s13048-016-0274-3

**Published:** 2016-10-20

**Authors:** Hossein Yazdekhasti, Zahra Rajabi, Soraya Parvari, Mehdi Abbasi

**Affiliations:** 1 Department of Anatomy, Faculty of Medicine, Tehran University of Medical Sciences, Tehran, Iran; 2 Department of Anatomy, Faculty of Medicine, Alborz University of Medical Sciences, Karaj, Iran

**Keywords:** Ovarian stem cells, Infertility, Oocyte, Immunosorting, Neo-oogenesis

## Abstract

Although existence of ovarian stem cells (OSCs) in mammalian postnatal ovary is still under controversy, however, it has been almost accepted that OSCs are contributing actively to folliculogenesis and neo-oogenesis. Recently, various methods with different efficacies have been employed for OSCs isolation from ovarian tissue, which these methods could be chosen depends on aim of isolation and accessible equipments and materials in lab. Although isolated OSCs from different methods have various traits and characterizations, which might become from their different nature and origin, however these stem cells are promising source for woman infertility treatment or source of energy for women with a history of repeat IVF failure in near future. This review has brought together and summarized currently used protocols for isolation and propagation of OSCs in vitro.

## Main text

**Fig. 1:**
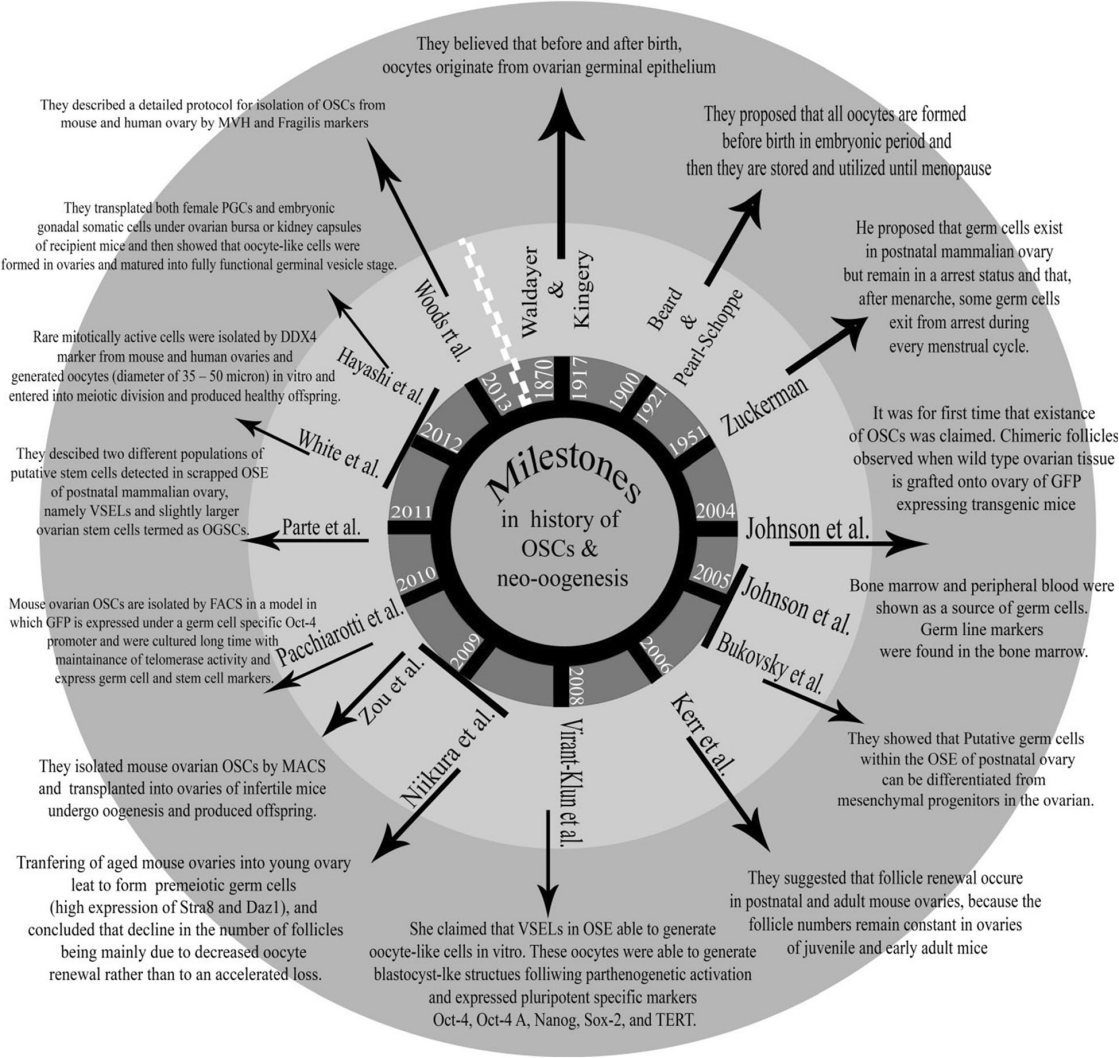
The OSCs discovery milestone. The old opinion about oogenesis was appeared from 1870 and this long standing opinion was maintained for many years until 2004 that Johnson et al. challenged it and after that many research groups reports their investigations on OSCs. In 2013, the final important research on isolation and propagation of OSCs was published by Prof. Tilly’s group

It had been widely accepted that mammalian females are endowed with a fixed number of oocytes and follicles at birth, but this long-standing dogma has been recently challenged. The Ovarian Stem cells (OSCs) discovery milestone has been shown in Fig. 1. By the end of 19th century and during the first half of the 20th century, two different opinions regarding oogenesis were raised. The first hypothesis was introduced by Waldeyer [1] in 1870 and followed by Kingery [2] who claimed that before and after birth, oocytes originate from germinal epithelium of ovary. The second hypothesis was firstly raised by Beard [3] in 1900 and elaborated by Pearl and Schoppe [4]. They proposed that all oocytes are formed before birth in embryonic period and then they are stored and utilized until menopause. Ultimately, in 1951 Lord Solomon Zuckerman published a paper and summarized all existing data at the time for and against presumption of postnatal neo-oogenesis [5]. This belief persisted constant and changeless until 2004 which finally by Professor Tilly’s group [6] this idea of fixed ovarian reserve was challenged and gates were opened for a more reliable and promising source for combating ovarian aging and keep the hopes alive for women who suffering from their infertility especially in the cases of Decrease Ovarian Reserve (DOR), Primary Ovarian Insufficiency (POI), Premature Ovarian Failure (POF) and age-associated ovarian dysfunction. Like some landmark discoveries which have accomplished in biology serendipitously, presence of OSCs in postnatal mammalian ovaries was recognized accidentally through oocyte-counting experiments in mice [6]. Meanwhile, kinetics experiments of declining in number of follicles through the life indicated that presence of OSCs is vital for supply of folliculogenesis during expected chronological lifespan [6, 7] and mathematical analysis shows that mouse ovaries are replenished with ~77 new follicles per day [6].

In spite of existing documents, there are three other evidences which confirm the theory of neo-oogenesis in postnatal mammalian ovaries, 1) germ cell–specific meiosis-commitment gene, stimulated by retinoic acid gene 8 (Stra8) [8], which is expressed highly in adult testes and in embryonic ovaries during the period of oogenesis [9] is rare but not absent in ovaries of reproductive-age mice [10, 11], 2) unilateral ovariectomy in female mice at first month of age accelerates the mitotic occurrence in oocytes of the remaining ovary 3 months later and 3) experiments showed that the number of traceable mitotic divisions in oocytes of aged mice exceeds those in younger counterparts [12, 13]. The best interpret for this evidence is that ovarian follicle pool is maintained during reproductive age and oocyte progenitor is contributed to oogenesis after birth [12, 14].

The phenomenon of postnatal neo-oogenesis and contribution of oogonial stem cells in reproductive activity in some species has been proven for many years such as teleost medaka [15] as well as *Drosophila* [16, 17], but there is not yet consensus among scientists regarding presence of OSCs in postnatal mammalian ovaries (Table 1).

**Table 1:** Critical approaches with pros and cons

Opinion	Cons	Pros
Johnson et al., 2004 [6] Johnson et al., 2005 [21] OSCs originate at a site extraneous to the ovary, namely the bone marrow, and are transported to the ovary via the circulatory system	Eggan, 2006 [56] By designing of a parabiotic mouse models showed that no evidence For bone marrow cells, or any other normally circulating cells, contribute to the formation of mature, ovulated oocytes.	Tilly et al., 2007 [57] He believed that Eggan focused solely on eggs retrieved from the oviducts following superovulation and did not include the outcome of evaluating the ovaries of their recipient mice for donor-derived immature oocytes.
	Begum, 2008 [58] No evidence was found to support the hypothesis that progenitor cells from extra-ovarian sources can repopulate the adult ovary. The findings are consistent with the conventional view that a limited number of oocytes are formed before birth and declines with age.	Lee et al., 2007 [59] They claimed that bone marrow transplantation rescued long-term fertility in CTx-treated females, but all offspring were derived from the recipient germline.
		Parte et al. [33, 37, 38] Bhartiya et al., [34–36] They believed that OSCs are originating from VSELs.
Johnson et al., 2004 [6] Bukovsky, 2005 [25] Johnson et al., 2005 [21] Zou et al., 2009 [18] OSCs are present in post natal mammalian ovary and are actively contributing in folliculogenesis and neo-oogenesis	Bristol-Gould et al., 2006 [60] By designing of a mathematical model of the dynamics of follicle progression, they indicated that no germline stem cells could be identified by SSEA-1 immunostaining.	Tilly et al., [61] Tilly et al., [57] Confirmed their previous results
	Malcolm Faddy, 2009 [62] Gosden et al., [63] They believed that new finding might be based on spurious results.	Kerr et al., [7] They found no evidence for ovarian germline stem cells, their data support the hypothesis of postnatal follicle renewal in postnatal and adult ovaries of C57BL/6 mice.
	Wallace [64] By mathematical modelling of the ovarian reserve found no evidence to support the occurrence of neo-oogenesis in humans	Abban et al., [65] They not only confirmed zou’s experimental results, but also they predict that FGSC arises between the border of PGC and oogonia development and the initiation of germline cysts.
	Byskov et al., [32] Using some histological and immunohistochemistry evaluations and based on previous observations claimed that the results presented by Johnson et al. (2004) [6] cannot support the concept of neo-oogenesis in the postnatal mouse ovary. Nor does there exist any evidence for neo-folliculogenesis in the adult mammalian ovary.	Others, [14, 19, 26, 44, 48, 50, 66–73]
	Liu et al. 2007 [74] They showed that We show that active meiosis, neo-oogenesis and GSCs are unlikely to exist in normal, adult, human ovaries. No early meiotic-specific or oogenesis-associated mRNAs for SPO11, PRDM9, SCP1, TERT and NOBOX were detectable in adult human ovaries using RT–PCR	
	Zhang et al., 2012 [75] By producing a multiple fluorescent Rosa26rbw/+;Ddx4-Cre germline reporter mouse model for in vivo and in vitro tracing of the development of female germline cell lineage, they showed that no mitotically active female germline progenitors exist in postnatal mouse ovaries	
	Lei et al., 2013 [76] Using sensitive lineage labeling system to determine whether stem cells are needed in female adult mice to compensate for follicular losses and to directly identify active germ-line stem cells, they showe that Female mice lack adult germ-line stem cells but sustain oogenesis using stable primordial follicles	
Johnson et al., 2004 [6] Positive BrdU Mitotic germ cells in ovarian epithelium	Elena Notarianni, 2011 [77] BrdU-incorporation arose from either mitochondrial (mt) DNA replication or DNA repair in oocytes, on the basis that “the degree of BrdU incorporation observed in cells due to either of these processes is several log orders less than that seen during replication of the nuclear genome during mitosis.	
Bukovsky and Virant Klun [25, 27] Cultured OSE gives rise to “oocyte-like” cells Mvh + germ cells located in the OSE “Oocyte-like” phenotype of cells in OSE derived cultures Other observation	Elena Notarianni, 2011 [77] These cells are actually undergoing apoptosis, necrosis or oncosis. Oocytes in transit across the OSE during exfoliation. Nondescript cells undergoing oncosis Reinterpretation of results by Notarianni	
White et al., 2012 [19] Woods et al. 2013 [20] Successful isolation of OSCs using DDX4 marker via FACS	Hernandez et al., 2015 [23] Zhang et al., 2015 [78] They were able to isolated a population of cells from the human ovarian cell preparation However, THEY did not detect any DDX4 mRNA expression by qPCR in these cells. They believed that the isolated cells bound tightly to the DDX4-specific antibody in FACS and became ‘DDX4-positive’ after culture. So use of the DDX4-specific antibody in FACS is not suitable for specifically selecting for a certain type of cell that expresses DDX4.	Park et al., 2014 [73] Woods et al., 2015 [22] Confirmed their previous results

However, after landmark discovery of presence of OSCs in postnatal mammalian ovaries started from 2004 by Professor Tilly’s group, different independent labs worldwide isolated and cultured OSCs by different methods and protocols which this review has brought together and summarized currently used protocols for isolation and propagation of OSCs in vitro. The strategy for selection of papers and interpreting results is based on the first article published and their fabulous findings in the field and to compare of traits of sorted cells, we tried to use papers which sorted cells and applied protocol for their isolation have been well characterized.

### FACS-based method

This method that has been used by Professor Tilly is based on immunological detection of a putative cell-surface variant of DEAD box polypeptide 4 (Ddx4) or so called Mouse vasa homolog (Mvh). Although Ddx4 is widely considered to be a cytoplasmic protein, but based on results from Ji Wu and colleagues [18], computer-based mapping of the Ddx4 and Ddx4 transmembrane-spanning domain, OSCs possess an externally putative extracellular epitope of Ddx4 which contributes to their isolation by Fluorescence-Activated Cell Sorting (FACS). The superiority of this method over others, even immunomagnetic sorting using the Ddx4 COOH antibody, is that only the FACS approach yields a viable and purified population of homogenous cells free from contaminating oocytes and non-germline cell lineages. For obtaining cells intended for FACS, ovaries from mice between 6 and 8 weeks of age and ovarian cortical tissue of women in their 20s, 30s, 40s and 50s were minced and enzymatically digested and passed through 70 μm filter to remove large tissue clumps and then through a 35 μm filter. Here, used antibody for isolation of OSCs was against the C terminus of Ddx4 and attained cells from human ovarian cortical tissue and mice ovaries were in range of 5–8 μm in diameter and identical in morphology and they had a genetic signature consistent with primitive germ cells. Also, after evaluation of teratoma-formation capacity of isolated OSCs, it was revealed that although OSCs express numerous stem cell and primitive germ cell markers, these cells have unique identity, distinct from the other types of pluripotent stem cells. The percent yield was 1.7 % ± 0.6 % (mean ± s.e.m.) Ddx4-positive as compared to the total viable cells sorted in human and 1.5 % ± 0.2 % in mice and by using value of genomic DNA content per cell, the incidence of OSCs per ovary was estimated 0.014 % ± 0.002 % in the mice, between 250 to slightly over 1000 viable Ddx4-positive cells from each young-adult mouse ovary after FACS of dispersates. Transplantation experiments of GFP-expressing OSCs into ovaries showed that not only OSCs had a stable integration into the ovaries and there were numerous follicles containing GFP-positive oocytes in recipient ovaries, but also they were able to produce GFP-positive embryo in in vivo study [13, 19, 20].

In vitro studies indicated that using feeder layer greatly facilitates the establishment of mouse and human OSCs in vitro, but is not vital for cell line establishment. Double positive for Ddx4 and BrdU dividing cell colonies were appeared after 10–12 weeks of culture in mouse (4–8 weeks in human) and results showed that estimated doubling time was 14 hours with required passage at confluence every 4–5 days (the proliferation rate was less in human OSCs which required passage at confluence every 7 days). 35–50 μm in diameter oocyte-like cells deduced by morphology and gene expression analyses were generated spontaneously from OSCs with maximum rate within 24–48 h after each passage in mice (the peak oocyte formation was at 72 h after each passage in human OSC cultures). Ultimately, successful establishment of cryopreserved and thawed human ovarian tissue samples in vitro already in development for females with cancer show that this method can be used as a novel approach in infertility treatment. In addition, this group has shown that bone marrow transplantation restored the oocyte production in wild-type sterilized mice by chemotherapy, as well as in ataxia telangiectasia mutated gene deficient mice, which are otherwise incapable of making oocytes, hence, They proposed that OSCs might be originated from peripheral blood and bone marrow [21].

In recent years, some publications showed that previously reported DDX4-positive OSCs that were purified from adult human and mouse ovaries using the DDX4-specific antibody are neither specific DDX4-expressing cells nor are they functional germline stem cells [22], they showed that polyclonal antibody specific to DDX4 (ab13840; Abcam) is entirely nonspecific and has a high affinity to attach to other cell types [23]. Therefore, more detailed studies are needed to fully characterizing these cells as OSCs.

### OSE Scraping method

This method was described for the first time in 2004 by Bukovsky group [24, 25] and then was used by Virant klun in order to isolation of OSCs from Ovarian Surface Epithelium (OSE) of the adult human ovaries with no naturally present oocytes and follicles [26]. Here, after scraping of OSE of postmenopausal and POF women, ovarian cells and fragments were subjected to density gradient centrifugation and then grown in vitro. In the OSE culture, putative stem cells proliferated and formed embryoid body-like structures [27]. Some cells grew intensively and became approximately 20 μm in diameter small and round oogonium like structures after 5–7 days and in extended condition (20 days), they reached to 95 μm diameter OLCs which are comparable to human oocytes in the in vitro fertilization program. They had genetic profile corresponding with oocyte (Oct-4A, Oct-4B, C-kit, VASA, and ZP2 transcription markers) and in some occasions, developed a zona pellucida-like structure around them. However, they did not expressed SCP3 marker as a meiosis initiation marker. This group also showed that generated OLCs are able to be activated and generate parthenogenetic blastocyst-like structures in vitro [27]. She believed that expression of C-kit in isolated cells is representative of their PGC ancestry and used a new term of embryonic-like stem cells of the adult for obtained cells by this method. She also believed that obtained putative OSCs could be compared to the Very Small Embryonic-Like Stem Cells (VSELs) found in different human and animal adult tissues and organs (bone marrow, bronchial epithelium, epidermis, myocardium, pancreas, and testes) as reported by Ratajczak et al. [28]. These stem cells were with a diameter from 3 to 5 μm, which is very comparable to the diameter of putative OSCs as well as in pattern of expression of SSEA-4 and Oct-4 transcription factor.

After histological and Flow-cytometry analysis of obtained cells, the proportion of putative stem cells was estimated up to 10 % just after scraping and after 20 days of culture this proportion increased to 32 %. These cells were slightly green colored, with a typical bubble-like structure and had large nuclei, which spread throughout the whole cell volume with a very small proportion of cytoplasm around them which in histological analysis often present among epithelial cells in the epithelial crypts, which extended into the ovarian cortex.

In addition, Virant klun and her collagenous in 2013, described two other methods for isolation of putative stem cells from OSE layer of reproductive-age, postmenopausal and POF women [29–31]. These two methods were FACS and Magnetic Activated Cell Sorting (MACS), based its on SSEA-4 surface antigen expression. The SSEA-4 positive cells made up to 1.6 % of the all cells in FACS method. Specimens from POF women were obtained by brushing of the ovarian cortex biopsies and after performing immunological isolation of SSEA-4 expressing stem cells, a similar, relatively homogenous population of small, SSEA-4-positive cells with diameters of up to 4 $\mu$m from the suspension of cells was attained [29]. She cultured putative stem cells for approximately 6 months in presence of follicular fluid and then different analysis showed that these cells expressed the analyzed markers of primordial germ cells (PRDM1, PRDM14, and DPPA3), pluripotency (OCT4A, SOX-2, SSEA-4, SALL4, CDH1, and LEFTY1) and some oocyte-specific markers (ZP3, SCP3, and c-KIT). Furthermore, microarray and Real-Time quantitative PCR (qPCR) analysis showed that putative ovarian stem cells and hESCs strongly expressed all analyzed genes (DPPA3, SALL4, CDH1, and LEFTY1) related to pluripotency and ESCs, while human adult fibroblasts (FBs) only weakly expressed these genes or did not express them at all and PGC related genes PRDM1 (BLIMP1) was highly expressed in small putative ovarian stem cells.

Antonin Bukovsky believes that functional mouse oocytes and sperm can be derived in vitro from somatic cell lines and also he claims that mesenchymal cells in the tunica albuginea of human and mouse ovary are bipotent progenitors with a commitment for both primitive granulosa and germ cells. He showed that after OSE scraping of human ovary and cultivation of OSE cells in presence of phenol red as mild estrogenic stimuli, OSE cells differentiated directly into large (180 μm) cells of the oocyte phenotype, while in absence of phenol red, they differentiated into small (15 μm) cells of granulosa phenotype, and epithelial, neural, and mesenchymal type cells. Furthermore, he indicated that not only primary follicles pool in adult human ovaries is not in static status, but also in order to elimination of spontaneous or environmentally induced genetic alterations of oocytes in resting primary follicles is in dynamic status [24, 25, 32].

Bhartiya and collagenous are another independent group who are working vastly on OSCs and evaluating effects of FSH on proliferation and differentiation potential of OSCs [33–38]. By scraping OSE method, they cultured OSE scraped cells from adult rabbit, sheep, monkey, and menopausal human OSE for 3 week period. Their results showed that there are two distinct populations of round Putative stem cells (PSCs) with different size. One population comprised 1–3 μm cells (smaller than RBCs) with DAPI positive and nuclear Oct-4 staining and SSEA-4 cell surface localization, whereas the second population was 4–7 μm in diameter with cytoplasmic Oct-4 and minimal cytoplasmic SSEA-4 and compacted heterochromatin [33]. They supposed that small PSCs are representing of pluripotent VSELs and bigger PSCs are immediate tissue committed progenitor stem cells derived from them. Also, epithelial cells in OSE scraped culture undergo epithelial-mesenchymal transition and give rise to somatic granulosa-like cells. They showed that OSCs can spontaneously differentiate into OLCs with prominent polar body-like protrusions and surrounded by distinct zona pellucida-like structure, even in some occasions, blastocyst-like structures with well-defined trophoectoderm and fluid-filled blastocoel-like structure with maximum diameter of 100–150 mm were observed. This group also demonstrated that in culture of ovarian scraped cells, FSH (0.5 IU/ml) and bFGF (100 ng/ml) affect OSCs proliferation and increase transition of primordial follicles to primary follicles [37]. Their results showed that both Follicle Stimulating Factor Receptor 1 (FSHR1) and FSHR3 mRNA were expressed in the OSCs but only FSHR3 mRNA was actively transcribed and expressed in the cytoplasm of OSCs after FSH treatment [34].

In recent work published by Bhartiya et al., they showed that chemotherapy led to complete loss of follicular reserve and cytoplasmic OCT-4 positive progenitors (ovarian germ stem cells) but VSELs survived. They claimed that after 6 days, MVH and GDF9 positive cells were present in OSE after chemotherapy and probably arising as a result of differentiation of the surviving VSELs [39]. They also indicated that after injection of 5-fluorouracil (5-FU) to mice and creation of stress model and hematopoiesis depletion in bone marrow, VSELs and hematopoietic stem cells (HSCs) were activated in response to the stress created by 5-FU in bone marrow and FSH could enhance hematopoietic recovery by at least 72 h. They claimed that both VSELs and HSCs expressed FSH receptors and FSH treatment enhanced hematopoietic recovery [40]. Meanwhile, in very recent interesting studies, Mierzejewska and collagenous relieved that VESLs possess receptors for FSH, LH, prolactin and androgen and these results support the concept of a potential developmental link between the germline and hematopoiesis [41, 42], so that this can be assumed that OSCs are originated from VSELs.

### MACS-based method

For the first time, Zou et al. reported successful isolation of Female Germline Stem Cells (FGSCs) from adults and 5 days old mice ovary by MACS using MVH marker and showed that after transfecting FGSCs with GFP and transplanting into sterile recipients, these cells restored fertility and recipients produced off normal and fertile offspring by natural mating with a wild-type C57BL/6 male [18]. After isolation and characterization of FGSCs, this group performed different assessment in order to confirmation of their potential and traits. Results of this study showed that presence of BrdU–MVH double positive cells in the ovarian surface epithelium, suggesting that they might be FGSCs. Isolated cells were very similar to freshly isolated type A Spermatogonial Stem Cells (SSCs) and they were Large round or ovoid cells with little cytoplasm, spherical nuclei with slight staining, a large ratio of nuclear plasma and nuclear diameter of 12–20 μm. Also this is important to notice that they were able to isolate just 200–300 cells from 9 to 12 neonatal mice and 50–100 cells from 6 to 8 adult mice. In culture, these cells proliferated and formed compact clusters of cells with blurred cell boundaries during just 7–8 passages. RT- PCR and immunocytochemical results showed that these cell express Oct4, MVH, Dazl, Blimp-1, Fragilis, Stella and Rex-1, whereas they did not express c-kit, Figla, Sox-2, Nanog, Scp1-3 or ZP3. Other results also demonstrated that after several passages, these cells had undifferentiated FGSC phenotypes, high telomerase activity, normal karyotype and positive alkaline phosphatase staining with weaker intensity in compared with ES cells. Ultimately, Combined Bisulphite Restriction Analysis (COBRA) showed that isolated cells had female imprinting pattern with partially methylated maternally imprinted region and demethylated paternally imprinted regions in FGSCs. Although in this study isolated cells have similar characteristics as same as SSCs, but there are some substantial differences between them including much less number of FGSCs in ovary, much slower growth of FGSCs during the initial phase of culture, FGSCs formed compact clusters of cells when they proliferated, whereas SSCs formed clumps and FGSCs have a female pattern and SSCs have a complete androgenic imprinting pattern.

In addition, this group designed a new study for improvement of their results in 2011 [43]. In this study, to optimize the purification of FGSCs, three different proteins expressed in germline cells were compared (CD9, Stpb-c and Fragilis) and their results showed that efficacy of FGSC purification from ovarian tissue by MACS method using fragilis marker was remarkably enhanced in compared with MVH that they had used in their previous study.

There is another study from this group in literature, in which by using same strategy (MACS with Fragilis marker) they isolated germline stem cells from post-natal rat ovary [44, 45]. In this study, just 200–300 cells were obtained from 20 ovaries and isolated cells were round with a high nuclear to cytoplasm ratio, and a size and morphology similar to those of mouse and freshly isolated type A spermatogonia. Dual immunofluorescence analysis of BrdU incorporation and Ddx4 expression was performed to confirmation of isolated fragilis^+^ cells were rat FGSCs. FGSCs formed spherical or grape-like clusters consisting of 4–8 cells during 3 weeks. After 10 weeks proliferation rate became rapid and cells required passaging at confluence every 5–6 days. In this study, after several passages, analysis showed that cells were positive for alkaline phosphatase with lower intensity and Oct4, Ddx4, Dazl, Blimp-1 and Fragilis were expressed in FGSCs, but no expression of Nanog, c-kit, Sox-2, Figla, Scp1-3 or Zp3.

In addition, Zhiyong et al. reported that they employed a new strategy to improve isolation and Identification of mouse oogonial stem cells [46]. They believed that two-step preparation including digestion and MACS would harm cells considering that the harvested cells from digested ovaries are in considerably small amount and purification would likely fail. Therefore, they cultured cell for 2–3 days directly after digestion which the total number of cells increased to 0.5–1 × 10^5^ and then, these cells were used for MACS by antibody of Fragilis. They showed that using this strategy, efficacy of the generation and characterization of OSCs significantly improved. They also showed that human umbilical cord mesenchymal stem cells as feeder will be useful and improve colony formation rate compared to STO feeder.

### ΔPE-Oct4-Gfp transgenic mice-based method

Nowadays, transgenic mice have become a major research resource, and applications of the transgenic approach have begun to infiltrate the world of biotechnology. Izadyar et al. in 2008, by using transgenic mice reported that they were able to isolate and generate multipotent cell lines from male gonads [47] and then in 2010 they used same strategy, using a transgenic mouse model in which GFP is expressed under a germ cell specific Oct-4 promoter, for isolation and derivation of germline stem cell from postnatal mouse ovary [48]. They isolated intended stem cells using GFP marker by FACS technique and cultured these isolated germline stem cells for almost one year. After this period, those did not lose their stemness characteristics, telomerase activity and normal karyotype. This group claimed that there are two distinct GFP-Oct-4 positive populations with different size and distribution in neonatal and adult mouse ovary. First population is in OSE region with the average diameter of 10–15 μm, and second population with the average diameter of 50–60 μm, located in the center of the follicles, representing oocytes. They believed that just 0.05 % all ovarian cells are GFP positive in adult, whereas in neonatal was 1–2 %, representing that the number of GFP positive cells reduced with advancing age. They also analyzed the ploidy of GFP-Oct-4 positive cells using propidium iodide and flow cytometry and reported that there is a population among GFP positive cells with germ cell and stem cell characteristics in which ploidy status was diploid. These cells were desired germline stem cells.

In this work, formed colonies from isolated cells after one week were round flat and some with a clear boundary and some appeared as a monolayer without a clear border. They also were stained positive for germ cell markers GCNA and c-Kit, pluripotent markers Oct-4, Nanog and GFR-a1, the receptor of GDNF, but they not formed malignancy after transplantation in recipients. After several passages, just some cells mainly in center of colonies became larger (up to 40 μm) and found OLC morphology surrounded by a layer of other cells resembling primordial follicle structures. In addition, they revealed that in presence of growth factors cocktail, OGSC differentiate into multiple lineages, whereas without growth factor, OLCs is formed with diameter of up to 60 μm. Moreover, different evaluations of in vitro-derived oocytes using Phalloidin, an actin cytoskeletal marker, and PNA, a marker for cortical granules showed that OLCs were surrounded by actin filaments and contained one diffuse nuclear chromatin and numerous of cortical granules throughout the cytoplasm as well as expressing early and late oocyte markers including Gdf9 and ZP1 even SCP3 using RT-PCR.

### Morphology based selection method

This approach was used for first time for isolation of male Germ Stem Cells (GSCs) from testis [49], then Abbasi’s group reported that they were able to isolate GSCs from ovary of mouse by morphology based selection method [50]. In this method, after enzymatic and mechanical digestion of ovaries, a pre-plating culture on gelatin coated dishes was done to eliminate fibroblast and somatic cells contamination. After 30 min pre-plating culture, buoyant cell were harvested and cultured on gelatin-coated 60 mm culture dishes. Embryonic-like colonies after 7–10 days were selected and mechanically removed using capillary pipette and transferred onto the inactivated Mouse Embryonic Fibroblasts (MEF) monolayer. Their molecular evaluations demonstrated that ovarian stem cell-like colonies were positive for alkaline phosphatase activity and expressed pluripotent and germ cell markers, such as Oct-4, Fragilis, Nanog, C-kit, Mvh, and Dazl and translation of genes to proteins were evaluated by Immunofluorescence of colonies against stem and germ cell specific markers such as Oct-4, Dazl, Mvh and SSEA1.

In continuation, they induced differentiation of OSCs into OLCs by co-culturing OSCs with granulosa cells for 11 days. Their results of immunofluorescence evaluations and RT-PCR showed that SCP3 and GDF9 were expressed in colonies, but pluripotency related genes were not expressed and cells did not show any significant growth in size (Parvari S, Yazdekhasti H, Rajabi Z, Gerayeli Malek V, Rastegar T, Abbasi M. Differentiation of mouse ovarian stem cells toward oocyte-like structure by co-culture with granulosa cells. Cell Reprogram. 2016. In press). This insignificant growth might be result of insufficient time course for differentiation induction or even maybe these cells were in their early developmental stage and need more time to reach appreciated size. Meanwhile, various growth factors and cytokines are needed to fully growth and differentiation of stem cells that due to fail of establishing correct cell to cell communication between ovarian stem cells and granulosa cells, these cells did not show any significant growth in size.

#### Future perspectives

Based on numerous papers that have being published each year regarding presence of OSCs in postnatal mammalian ovary and their capacity to produce oocytes in vivo and in vitro, so it can be deducted that neo-oogenesis is gradually accepted by reproductive biology area. However, some important questions remain elusive from both basic science and clinical perspectives which have to be cleared in future investigations before advent of therapeutic procedures for clinical management of the ovarian reserve and fertility as well as treatment of infertility. What is the potential role of OSCs in postnatal ovary? What is origin of OSCs? Where is their exact localization in ovary (cortex or surface epithelium)? What is the optimized method for their isolation from ovary? What is the constitution of OSCs niche and which kind of factors are secreted in microenvironment? These are some question that time will tell us presumptive answers. The area of mammalian OSCs has been initiated for less than 17 years, in compared with spermatogonial stem cells stared from 1960s [51, 52], however researches in this area are ongoing and time will tell us which method could be optimal for use in clinical practice to treat infertility in young women.

## Conclusion

In this review, we tried to summarize all used protocols by independent research lab world widely regarding OSCs isolation from ovary in different species (Table 2). There are some other reviews in this area [53–55], but we have tried to bring detailed characterizations of ovarian stem cells. This survey displays that isolated putative stem cells have different sizes with various characterizations, in which used markers for their isolation are different as well. These differences might be originated from their different nature, origin or potential roles in ovary. It is even possible that cells are in different developmental stage or these cells have another identity (c-KIT has been expressed in some studies [26, 27, 29, 30], whereas in some others not [18, 43–45]. Each protocol has own advantages and disadvantages that it is recommended before starting each protocol, aim of isolation and accessible equipments and materials in lab be considered.

**Table 2:** Summarization of all used protocols for isolation of germline stem cells from different species ovary (NS: Not Stated)

Method	Author year & reference	Species (s)	Age	Marker	OSC traits	OLC size	Culture duration	Putative OSC’s origin	Colony shape and formation duration	Superiority and limitation	Growth factors used
FACS	White 2012 [6, 19, 20]	Human Mouse (C57BL/6)	6–8 weeks mice 20s, 30s, 40s, 50s women	COOH–DDX4	5–8 μm primitive germ cells genetic signature	35–50 μm	18 months	Bone Marrow and peripheral blood	10–12 weeks in mouse 4–8 weeks in human	Viable and purified population	N-2 supplement, LIF EGF bFGF GDNF
	Virant klun 2008 [29, 30]	Human	Reproductive ages and menopause	SSEA-4	2–4 μm and bigger cells 8 μm small, round and yellow-color	60 μm	21–23 days	an integral part of the ovarian surface epithelium	approximately 3 months	Homogenous population	Without growth factors
	Dunlop 2014. [79]	Bovine Human	NS	DDX4	NS	NS	several month	NS	NS	NS	NS
OSE Scraping	Virant klun 2008 [23, 24]	Human	Reproductive ages and menopause	OSCs separated by density gradient centrifugation	Small round cells with a bubble-like structure	95 μm	20 days	PGCs and VSELs	NS	Simple and easy	Without growth factors
	Bukovsky 2004 [21, 22]	Human	27–38 years women	Whole ovarian cells were cultured	10 μm nuclear MAPK & PS1 immunoexpression	180 μm	5–6 days	mesenchymal Somatic cells in the tunica albuginea	NS	Simple and easy	Without growth factors
	Bhartiya 2011 [33]	Human Monkey Sheep Rabbit	Menopausal women with a mean age range of 46 years	Whole ovarian cells were cultured	Smaller PSCs: 1–3 μm Larger PSCs: 4–7 μm Dark, bubbly, shiny appearance Large, darkly stained nuclei with a thin rim of cytoplasm	130 μm	3 weeks	VSELs origin	Day 10 Flat with a well-defined margin ES cell-like colonies 3-dimensional dense floating embryoid body-like structures	Simple and easy	Without growth factors
MACS	Johnson 2005 [21]	Mouse	6 and 9 weeks	SSEA-1	Isolated cells were used directly for RT-PCR			Bone Marrow and peripheral blood	NS	NS	NS
	Zou 2009 [18]	Mouse (C57BL/6)	Adult and 5 day old female	MVH	Large round or ovoid cells with little cytoplasm and spherical nuclei with slight staining, a large ratio of nuclear plasma and nuclear diameter of 12–20 μm	NS	15 months and more for neonatal FGSCs 6 months for adults FGSCs	NS	After 7–8 passages (forming cluster)	Poor describing details Relatively low efficiency	LIF Transferrin Insulin Putrescine EGF GDNF bFGF
	Zou 2011 [43]	CD-1 mice	Fragilis	5-day-old female	NS	NS	NS	NS	NS	Improved Efficiency	LIF Transferrin Insulin Putrescine EGF GDNF bFGF
	Zhou 2013 [38, 39]	Rat	5-day-old	Fragilis	Round with a high nuclear to cytoplasm ratio	55–60 um	More than one year	NS	In first 3 weeks: spherical or grape-like clusters consisting of 4–8 cells	Better efficiency of gene transfer for producing transgenic rat	LIF EGF GDNF bFGF
					size and morphology similar to mouse FGSCs						
	Bui 2014 [69]	Pig	Prepubertal gilts	SSEA-4	5–7 μm Completely round nuclei that took up almost the entire volume of the cell	>100 μm	6 months	VSELs and epiblast-derived PGCs	Spherical colonies comprising compact clusters of small round after 1 day Dark and shiny colonies	Better culture condition for establishment and long-term maintenance of PSCs	B27 SCF
Using ΔPE-Oct4-Gfp transgenic mice	Pacchiarotti 2010 [48]	Mouse	2 and 5 days old or one adult mouse	GFP which is express under Oct-4 promoter	10–15 μm Located in OSE positive for VASA, c-Kit, SSEA-10.05 % GFP positive in adults and 1–2 % in neonata	Up to 60 μm	More than one year	They are reserved pool in the quiescent state	One week	Is not optimal for several reasons [20]	Insulin Transferrin Selenium Fibronectin
Morphology based selection methods	Parvari 2015 [50]	Mouse	5–7 day old	OSCs separated by colony selection	2–4 μm As same as VSELs	Slightly bigger than OSCs	11 days	VSELs origin	4 days after pre-plating Small colonies were highly compact without a clear border	Easy Affordable	LIF

What is certain and undeniable, presence of ovarian stem cell in postnatal mammalian ovaries, but its contribution in neo-oogenesis phenomenon is under question. Some believe that in aged ovaries, stem cell niches fail to support stem cell division and stem cells are in quiescent state [10] and some others believe that these stem cells are actively contribute to follicle formation [6, 21], however, this needs more investigation to be figured out.

Based on results from several approaches, transplantation of ovarian stem cells into recipients could restore fertility and produce live offspring [18, 19]. This means that there is a promising cure in near future for infertile women who their ovaries are without follicle or oocyte. Although, some believe that OSCs still have not external existence, but with respect to their opinion, this requires many more years of study to fully grasp the importance of OSCs in reproductive biology.

## Abbreviations

COBRA: Combined bisulphite restriction analysis
Ddx4: DEAD box polypeptide 4
DOR: Decrease ovarian reserve
FACS: Fluorescence-activated cell sorting
FGSCs: Female germline stem cells
GSCs: Germ stem cells
HSCs: hematopoietic stem cells
MACS: Magnetic activated cell sorting
MEF: Mouse embryonic fibroblasts
Mvh: Mouse vasa homolog
OSCs: Ovarian stem cells
OSE: Ovarian surface epithelium
POF: Premature ovarian failure
POI: Primary ovarian insufficiency
PSCs: Putative stem cells
SSCs: Spermatogonial stem cells
Stra8: Stimulated by retinoic acid gene 8
VSELs: Very Small embryonic-like stem cells

## References

[1] Waldeyer W. Eierstock und Ei. Leipzig, W. Engelmann, 1870.

[2] Kingery HM. Oogenesis in the white mouse. J Morphol. 1917;30:261–315. doi:10.1002/jmor.10503001081917.

[3] Beard J (1900). The morphological continuity of the germ cells in Raja batis. Anat Anz.

[4] Pearl R, Schoppe WF (1921). Studies on the physiology of reproduction in the domestic fowl. J Exp Zool.

[5] Zuckerman S (1951). The number of oocytes in the mature ovary. Recent Prog Horm Res.

[6] Johnson J, Canning J, Kaneko T, Pru JK, Tilly JL (2004). Germline stem cells and follicular renewal in the postnatal mammalian ovary. Nature.

[7] Kerr JB, Duckett R, Myers M, Britt KL, Mladenovska T (2006). Quantification of healthy follicles in the neonatal and adult mouse ovary: evidence for maintenance of primordial follicle supply. Reproduction.

[8] Oulad-Abdelghani M, Bouillet P, Décimo D, Gansmuller A, Heyberger S (1996). Characterization of a premeiotic germ cell-specific cytoplasmic protein encoded by Stra8, a novel retinoic acid-responsive gene. J Cell Biol.

[9] Bowles J, Koopman P (2007). Retinoic acid, meiosis and germ cell fate in mammals. Development.

[10] Niikura Y, Niikura T, Tilly JL (2009). Aged mouse ovaries possess rare premeiotic germ cells that can generate oocytes following transplantation into a young host environment. Aging (Albany NY).

[11] Wang N, Tilly JL (2010). Epigenetic status determines germ cell meiotic commitment in embryonic and postnatal mammalian gonads. Cell Cycle.

[12] Reizel Y, Itzkovitz S, Adar R, Elbaz J, Jinich A (2012). Cell lineage analysis of the mammalian female germline. PLoS Genet.

[13] Woods DC, White YAR, Tilly JL (2013). Purification of oogonial stem cells from adult mouse and human ovaries: an assessment of the literature and a view toward the future. Reprod Sci.

[14] Woods DC, Telfer EE, Tilly JL (2012). Oocyte family trees: old branches or new stems. PLoS Genet.

[15] Nakamura S, Kobayashi K, Nishimura T, Higashijima S-i, Tanaka M (2010). Identification of germline stem cells in the ovary of the teleost medaka. Science.

[16] Wong MD, Jin Z, Xie T (2005). Molecular mechanisms of germline stem cell regulation. Annu Rev Genet.

[17] Waskar M, Li Y, Tower J (2005). Stem cell aging in the Drosophila ovary. Age.

[18] Zou K, Yuan Z, Yang Z, Luo H, Sun K (2009). Production of offspring from a germline stem cell line derived from neonatal ovaries. Nat Cell Biol.

[19] White YA, Woods DC, Takai Y, Ishihara O, Seki H (2012). Oocyte formation by mitotically active germ cells purified from ovaries of reproductive-age women. Nat Med.

[20] Woods DC, Tilly JL (2013). Isolation, characterization and propagation of mitotically active germ cells from adult mouse and human ovaries. Nat Protoc.

[21] Johnson J, Bagley J, Skaznik-Wikiel M, Lee H-J, Adams GB (2005). Oocyte generation in adult mammalian ovaries by putative germ cells in bone marrow and peripheral blood. Cell.

[22] Woods DC, Tilly JL (2015). Reply to Adult human and mouse ovaries lack DDX4-expressing functional oogonial stem cells. Nat Med.

[23] Hernandez SF, Vahidi NA, Park S, Weitzel RP, Tisdale J (2015). Characterization of extracellular DDX4-or Ddx4-positive ovarian cells. Nat Med.

[24] Bukovsky A, Caudle MR, Svetlikova M, Upadhyaya NB (2004). Origin of germ cells and formation of new primary follicles in adult human ovaries. Reprod Biol Endocrinol.

[25] Bukovsky A, Svetlikova M, Caudle MR (2005). Oogenesis in cultures derived from adult human ovaries. Reprod Biol Endocrinol.

[26] Virant-Klun I, Zech N, Rožman P, Vogler A, Cvjetičanin B (2008). Putative stem cells with an embryonic character isolated from the ovarian surface epithelium of women with no naturally present follicles and oocytes. Differentiation.

[27] Virant-Klun I, Rozman P, Cvjeticanin B, Vrtacnik-Bokal E, Novakovic S (2009). Parthenogenetic embryo-like structures in the human ovarian surface epithelium cell culture in postmenopausal women with no naturally present follicles and oocytes. Stem Cells Dev.

[28] Ratajczak M, Machalinski B, Wojakowski W, Ratajczak J, Kucia M (2007). A hypothesis for an embryonic origin of pluripotent Oct-4&plus; stem cells in adult bone marrow and other tissues. Leukemia.

[29] Virant-Klun I, Skutella T, Hren M, Gruden K, Cvjeticanin B (2013). Isolation of small SSEA-4-positive putative stem cells from the ovarian surface epithelium of adult human ovaries by two different methods. Biomed Res Int.

[30] Virant-Klun I, Skutella T, Kubista M, Vogler A, Sinkovec J (2013). Expression of pluripotency and oocyte-related genes in single putative stem cells from human adult ovarian surface epithelium cultured in vitro in the presence of follicular fluid. Biomed Res Int.

[31] Virant-Klun I, Stimpfel M, Cvjeticanin B, Vrtacnik-Bokal E, Skutella T (2013). Small SSEA-4-positive cells from human ovarian cell cultures: related to embryonic stem cells and germinal lineage. J Ovarian Res.

[32] Byskov AG, Faddy MJ, Lemmen JG, Andersen CY (2005). Eggs forever?. Differentiation.

[33] Parte S, Bhartiya D, Telang J, Daithankar V, Salvi V (2011). Detection, characterization, and spontaneous differentiation in vitro of very small embryonic-like putative stem cells in adult mammalian ovary. Stem Cells Dev.

[34] Bhartiya D, Parte S, Patel H, Sriraman K, Zaveri K (2015). Novel action of FSH on stem cells in adult mammalian ovary induces postnatal oogenesis and primordial follicle assembly. Stem Cells International.

[35] Bhartiya D, Sriraman K, Gunjal P, Modak H (2012). Gonadotropin treatment augments postnatal oogenesis and primordial follicle assembly in adult mouse ovaries. J Ovarian Res.

[36] Bhartiya D, Sriraman K, Parte S (2012). Stem cell interaction with somatic niche may hold the key to fertility restoration in cancer patients. Obstet Gynecol Int.

[37] Parte S, Bhartiya D, Manjramkar DD, Chauhan A, Joshi A (2013). Stimulation of ovarian stem cells by follicle stimulating hormone and basic fibroblast growth factor during cortical tissue culture. J Ovarian Res.

[38] Parte S, Bhartiya D, Patel H, Daithankar V, Chauhan A (2014). Dynamics associated with spontaneous differentiation of ovarian stem cells in vitro. J Ovarian Res.

[39] Sriraman K, Bhartiya D, Anand S, Bhutda S (2015). Mouse Ovarian Very Small Embryonic-Like Stem Cells Resist Chemotherapy and Retain Ability to Initiate Oocyte-Specific Differentiation. Reprod Sci.

[40] Shaikh A, Bhartiya D, Kapoor S, Nimkar H (2016). Delineating the effects of 5-fluorouracil and follicle-stimulating hormone on mouse bone marrow stem/progenitor cells. Curr Stem Cell Res Ther.

[41] Mierzejewska K, Borkowska S, Suszynska E, Suszynska M, Poniewierska-Baran A (2015). Hematopoietic stem/progenitor cells express several functional sex hormone receptors—novel evidence for a potential developmental link between hematopoiesis and primordial germ cells. Stem Cells Dev.

[42] Abdelbaset‐Ismail A, Suszynska M, Borkowska S, Adamiak M, Ratajczak J (2016). Human haematopoietic stem/progenitor cells express several functional sex hormone receptors. J Cell Mol Med.

[43] Zou K, Hou L, Sun K, Xie W, Wu J (2011). Improved efficiency of female germline stem cell purification using fragilis-based magnetic bead sorting. Stem Cells Dev.

[44] Zhang Y, Yang Z, Yang Y, Wang S, Shi L (2011). Production of transgenic mice by random recombination of targeted genes in female germline stem cells. J Mol Cell Biol.

[45] Zhou L, Wang L, Kang JX, Xie W, Li X (2014). Production of fat-1 transgenic rats using a post-natal female germline stem cell line. Mol Hum Reprod.

[46] Lu Z, Wu M, Zhang J, Xiong J, Cheng J (2015). Improvement in Isolation and Identification of Mouse Oogonial Stem Cells. Stem Cells Int.

[47] Izadyar F, Pau F, Marh J, Slepko N, Wang T (2008). Generation of multipotent cell lines from a distinct population of male germ line stem cells. Reproduction.

[48] Pacchiarotti J, Maki C, Ramos T, Marh J, Howerton K (2010). Differentiation potential of germ line stem cells derived from the postnatal mouse ovary. Differentiation.

[49] Guan K, Wolf F, Becker A, Engel W, Nayernia K (2009). Isolation and cultivation of stem cells from adult mouse testes. Nat Protoc.

[50] Parvari S, Abbasi M, Abbasi N, Malek VG, Amidi F (2015). Stem cell isolation by a morphology-based selection method in postnatal mouse ovary. Arch Med Sci.

[51] Clermont Y, Bustos‐Obregon E (1968). Re‐examination of spermatogonial renewal in the rat by means of seminiferous tubules mounted “in toto”. Am J Anat.

[52] Schulze C (1979). Morphological characteristics of the spermatogonial stem cells in man. Cell Tissue Res.

[53] Silvestris E, D’Oronzo S, Cafforio P, D’Amato G, Loverro G (2015). Perspective in infertility: the ovarian stem cells. J Ovarian Res.

[54] Pan Z, Sun M, Liang X, Li J, Zhou F (2015). The controversy, challenges, and potential benefits of putative female Germline stem cells research in mammals. Stem Cells Int.

[55] Virant-Klun I (2015). Postnatal oogenesis in humans: a review of recent findings. Stem Cells Cloning.

[56] Eggan K, Jurga S, Gosden R, Min IM, Wagers AJ (2006). Ovulated oocytes in adult mice derive from non-circulating germ cells. Nature.

[57] Tilly JL, Niikura Y, Rueda BR (2009). The current status of evidence for and against postnatal oogenesis in mammals: a case of ovarian optimism versus pessimism?. Biol Reprod.

[58] Begum S, Papaioannou V, Gosden R (2008). The oocyte population is not renewed in transplanted or irradiated adult ovaries. Hum Reprod.

[59] Lee H-J, Selesniemi K, Niikura Y, Niikura T, Klein R (2007). Bone marrow transplantation generates immature oocytes and rescues long-term fertility in a preclinical mouse model of chemotherapy-induced premature ovarian failure. J Clin Oncol.

[60] Bristol-Gould SK, Kreeger PK, Selkirk CG, Kilen SM, Mayo KE (2006). Fate of the initial follicle pool: empirical and mathematical evidence supporting its sufficiency for adult fertility. Dev Biol.

[61] Tilly JL, Johnson J (2007). Recent arguments against germ cell renewal in the adult human ovary: is an absence of marker gene expression really acceptable evidence of an absence of oogenesis?. Cell Cycle.

[62] Faddy M, Gosden R (2009). Let’s not ignore the statistics. Biol Reprod.

[63] Gosden R, Telfer E, Faddy M. Germ line stem cells and adult ovarian function. Stem Cells Human Reprod. 2009:57–68.

[64] Wallace WHB, Kelsey TW (2010). Human ovarian reserve from conception to the menopause. PLoS One.

[65] Abban G, Johnson J (2009). Stem cell support of oogenesis in the human. Hum Reprod.

[66] Bukovsky A (2011). Ovarian stem cell niche and follicular renewal in mammals. Anat Rec.

[67] Woods DC, Tilly JL (2012). The next (re) generation of ovarian biology and fertility in women: is current science tomorrow’s practice?. Fertil Steril.

[68] Khosravi-Farsani S, Amidi F, Roudkenar MH, Sobhani A (2015). Isolation and enrichment of mouse female germ line stem cells. Cell Journal (Yakhteh).

[69] Bui H-T, Van Thuan N, Kwon D-N, Choi Y-J, Kang M-H (2014). Identification and characterization of putative stem cells in the adult pig ovary. Development.

[70] De Felici M (2010). Germ stem cells in the mammalian adult ovary: considerations by a fan of the primordial germ cells. Mol Hum Reprod.

[71] Hu Y, Bai Y, Chu Z, Wang J, Wang L (2012). GSK3 inhibitor‐BIO regulates proliferation of female germline stem cells from the postnatal mouse ovary. Cell Prolif.

[72] Zhou L, Wang L, Kang JX, Xie W, Li X, et al. Production of fat-1 transgenic rats using a post-natal female germline stem cell line. Mol Hum Reprod. 2014;gat081. doi:10.1093/molehr/gat081. 10.1093/molehr/gat081 24258451

[73] Park ES, Tilly JL (2015). Use of DEAD-box polypeptide-4 (Ddx4) gene promoter-driven fluorescent reporter mice to identify mitotically active germ cells in post-natal mouse ovaries. Mol Hum Reprod.

[74] Liu Y, Wu C, Lyu Q, Yang D, Albertini DF (2007). Germline stem cells and neo-oogenesis in the adult human ovary. Dev Biol.

[75] Zhang H, Zheng W, Shen Y, Adhikari D, Ueno H (2012). Experimental evidence showing that no mitotically active female germline progenitors exist in postnatal mouse ovaries. Proc Natl Acad Sci.

[76] Lei L, Spradling AC (2013). Female mice lack adult germ-line stem cells but sustain oogenesis using stable primordial follicles. Proc Natl Acad Sci.

[77] Notarianni E (2011). Reinterpretation of evidence advanced for neo-oogenesis in mammals, in terms of a finite oocyte reserve. J Ovarian Res.

[78] Zhang H, Panula S, Petropoulos S, Edsgärd D, Busayavalasa K (2015). Adult human and mouse ovaries lack DDX4-expressing functional oogonial stem cells. Nat Med.

[79] Dunlop CE, Bayne RA, McLaughlin M, Telfer EE, Anderson RA (2014). Isolation, purification, and culture of oogonial stem cells from adult human and bovine ovarian cortex. Lancet.

